# Organ donation after out-of-hospital cardiac arrest: a population-based study of data from the Paris Sudden Death Expertise Center

**DOI:** 10.1186/s13613-022-01023-7

**Published:** 2022-06-06

**Authors:** M. Renaudier, Y. Binois, F. Dumas, L. Lamhaut, F. Beganton, D. Jost, J. Charpentier, O. Lesieur, E. Marijon, X. Jouven, A. Cariou, W. Bougouin, Paris Sudden Death Expertise Center group, F. Adnet, J. M. Agostinucci, N. Aissaoui-Balanant, V. Algalarrondo, F. Alla, C. Alonso, W. Amara, D. Annane, C. Antoine, P. Aubry, E. Azoulay, F. Beganton, C. Billon, W. Bougouin, J. Boutet, C. Bruel, P. Bruneval, A. Cariou, P. Carli, E. Casalino, C. Cerf, A. Chaib, B. Cholley, Y. Cohen, A. Combes, J. M. Coulaud, M. Crahes, D. Da Silva, V. Das, A. Demoule, I. Denjoy, N. Deye, J. L. Diehl, S. Dinanian, L. Domanski, D. Dreyfuss, D. Duboc, J. L. Dubois-Rande, F. Dumas, J. Duranteau, J. P. Empana, F. Extramiana, J. Y. Fagon, M. Fartoukh, F. Fieux, M. Gabbas, E. Gandjbakhch, G. Geri, B. Guidet, F. Halimi, P. Henry, F. Hidden Lucet, P. Jabre, L. Joseph, D. Jost, X. Jouven, N. Karam, H. Kassim, J. Lacotte, K. Lahlou-Laforet, L. Lamhaut, A. Lanceleur, O. Langeron, T. Lavergne, E. Lecarpentier, A. Leenhardt, N. Lellouche, V. Lemiale, F. Lemoine, F. Linval, T. Loeb, B. Ludes, C. E. Luyt, A. Maltret, N. Mansencal, N. Mansouri, E. Marijon, J. Marty, E. Maury, V. Maxime, B. Megarbane, A. Mekontso-Dessap, H. Mentec, J. P. Mira, X. Monnet, K. Narayanan, N. Ngoyi, M. C. Perier, O. Piot, R. Pirracchio, P. Plaisance, B. Plaud, I. Plu, J. H. Raphalen, M. Raux, F. Revaux, J. D. Ricard, C. Richard, B. Riou, F. Roussin, F. Santoli, F. Schortgen, A. Sharifzadehgan, T. Sharshar, G. Sideris, T. Similowski, C. Spaulding, J. L. Teboul, J. F. Timsit, J. P. Tourtier, P. Tuppin, C. Ursat, O. Varenne, A. Vieillard-Baron, S. Voicu, K. Wahbi, V. Waldmann

**Affiliations:** 1 Paris Cardiovascular Research Center (PARCC), INSERM Unit 970, Paris, France; 2 Paris Sudden Death Expertise Center, Paris, France; 3 Emergency Department, Cochin-Hotel-Dieu Hospital, AP-HP, Paris, France; 4 Université de Paris, Paris, France; 5 Intensive Care Unit and SAMU 75, Necker Enfants-Malades Hospital, Paris, France; 6 Brigade Des Sapeurs-Pompiers de Paris (BSPP), Paris, France; 7 Medical Intensive Care Unit, AP-HP, Cochin Hospital, Paris Cedex 14, France; 8 Intensive Care Unit, Saint Louis General Hospital, La Rochelle, France; 9 Cardiology Department, European Georges Pompidou Hospital, AP-HP, Paris, France; 10 Ramsay Générale de Santé, Hôpital Privé Jacques Cartier, Massy, France

**Keywords:** Out-of-hospital cardiac arrest, Epidemiology, Organ donation, Brain death

## Abstract

**Background:**

Organ shortage is a major public health issue, and patients who die after out-of-hospital cardiac arrest (OHCA) could be a valuable source of organs. Here, our objective was to identify factors associated with organ donation after brain death complicating OHCA, in unselected patients entered into a comprehensive real-life registry covering a well-defined geographic area.

**Methods:**

We prospectively analyzed consecutive adults with OHCA who were successfully resuscitated, but died in intensive care units in the Paris region in 2011–2018. The primary outcome was organ donation after brain death. Independent risk factors were identified using logistic regression analysis. One-year graft survival was assessed using Cox and log-rank tests.

**Results:**

Of the 3061 included patients, 136 (4.4%) became organ donors after brain death, i.e., 28% of the patients with brain death. An interaction between admission pH and post-resuscitation shock was identified. By multivariate analysis, in patients with post-resuscitation shock, factors associated with organ donation were neurological cause of OHCA (odds ratio [OR], 14.5 [7.6–27.4], *P* < 0.001), higher pH (OR/0.1 increase, 1.3 [1.1–1.6], *P* < 0.001); older age was negatively associated with donation (OR/10-year increase, 0.7 [0.6–0.8], *P* < 0.001). In patients without post-resuscitation shock, the factor associated with donation was neurological cause of OHCA (OR, 6.9 [3.0–15.9], *P* < 0.001); higher pH (OR/0.1 increase, 0.8 [0.7–1.0], *P* = 0.04) and OHCA at home (OR, 0.4 [0.2–0.7], *P* = 0.006) were negatively associated with organ donation. One-year graft survival did not differ according to Utstein characteristics of the donor.

**Conclusions:**

4% of patients who died in ICU after OHCA led to organ donation. Patients with OHCA constitute a valuable source of donated organs, and special attention should be paid to young patients with OHCA of neurological cause.

**Supplementary Information:**

The online version contains supplementary material available at 10.1186/s13613-022-01023-7.

## Background

Organ shortage is a major public health issue. In 2020, 39,000 organs were transplanted in the United States, while 107,000 patients were on organ donation waiting lists [1]. Finding new sources of organs is a crucial concern. Patients who die after out-of-hospital cardiac arrest (OHCA) may constitute a valuable pool of organ donors. Each year, 300,000 and 40,000 patients experience OHCA in the United States and France, respectively [2]. Over 90% of these patient die, either before hospital admission or in-hospital [3–5]. Among patients admitted alive to hospital, 10% to 15% progress to brain death [6]. Few patients become organ donors after OHCA [6–9].

Organ viability does not differ between donors who die after cardiac arrest and other donors: Sandroni et al. reported a similar 1-year survival rate of kidneys, livers and hearts between cardiac arrest donors and other donors [10]. Similarly, Orioles et al. did not report any significant difference in graft survival (kidney, heart, liver) between organs grafted after cardiac arrest or non-cardiac arrest [11]. Finally, West et al. reported similar results regarding heart, lung, kidney and liver [12]. Knowledge of factors associated with organ donation might help to identify potential donors, thus improving their management in intensive care units (ICUs), based on guidelines [13, 14]. In two retrospective studies [15, 16], age, sex, initial rhythm, bystander and epinephrine use were associated with organ donation. Treatment with extracorporeal membrane oxygenation (ECMO) might be also associated with organ donation [17]. More generally, better understanding of organ donation after OHCA may increase the number of transplanted organs.

The objective of this study was to describe the frequency and predictors of organ donation after brain death in patients admitted to ICUs after OHCA. To this end, we used a multicenter prospective population-based registry.

## Methods

The study methodology is consistent with the Strengthening the Reporting of Observational studies in Epidemiology guidelines [18].

### Setting, population, and donation process

The Sudden Death Expertise Center (SDEC) Registry is a population-based prospective registry of cases of OHCA in Paris, France, and three of its suburbs (Hauts-de-Seine, Seine-Saint-Denis, Val-de-Marne). The population covered is about 6.8 million on 762 km^2^ (294 mi^2^). The registry was started in May 2011. In France, the first responders for patients with OHCA are mobile emergency units and fire squadrons. On-scene resuscitation is delivered by a team that includes at least one physician trained in emergency medicine according to international guidelines [19]. Patients in whom the return of spontaneous circulation (ROSC) is achieved are then referred to the ICU.

All adults (age > 18 years) who experienced OHCA in the registry area were included in the SDEC. For the present study, we included all registry patients who were admitted alive to ICUs from May 15, 2011, to December 31, 2018, and who died before ICU discharge. Only exclusion criteria were unavailability of the medical report and donation after circulatory death (Maastricht III procedure) [20]. Donation after circulatory death was relatively new and rare in France during the study period and could only be performed in very selected hospitals. On the one hand, these patients did not fulfill brain death criteria, and on the other, they could not be considered as non-donors. Therefore, considering the scarcity of cases (< 0.5% of patients screened), we chose to exclude them.

In France, brain death is a clinical and legal diagnosis, as previously described [21–23]. In France, care can be continued pending brain death determination, but when a decision of withdrawal of life-sustaining therapy is made, patients are no longer suitable for a process of donation after brain death. Clinical diagnosis of brain death must be performed in the absence of confounding factors (sedation, hypothermia), and is based on deep coma, loss of all brainstem reflexes, with an apnea test. Ancillary tests (CT-scan or electroencephalogram) are used to confirm diagnosis before organ donation is allowed. Medical staff must report patients with brain death to the *Agence de la Biomédecine*, the national organization that handles procedures involving cells, tissues, and organs. An organ-procurement coordinator then completes a detailed evaluation of the patient to determine suitability for organ donation. Exclusion criteria include unknown patient identity, active malignancy, multisystem organ failure, and active viral infection (HIV or human T-cell lymphotropic virus) [24]. The coordinator checks that eligible patients are not on the organ-donation opt-out list and that the patient’s health surrogate has no knowledge of any unwillingness of the patient to become an organ donor.

### Data collection

Demographic data, comorbidities, location and cause of OHCA, and other OHCA characteristics according to the Utstein style were recorded prospectively in the electronic SDEC Registry [25]. Causes of OHCA were dichotomized as neurological or other. OHCA were considered of neurological cause in case of an acute neurological mechanism responsible for cardiac arrest: subarachnoid hemorrhage, ischemic stroke, intracerebral hemorrhage, traumatic brain injury, sub/epidural hematoma, or cerebral thrombophlebitis [26, 27]. Prehospital management data included presence of a bystander, bystander cardiopulmonary resuscitation (CPR), shockable rhythm before advanced life support, epinephrine delivery and total dose given on-site during advanced life support, no-flow time (from OHCA to CPR initiation), and low-flow time (from CPR initiation to ROSC). We classified admitting hospitals as transplant or non-transplant centers. Hospital management data included admission pH, post-resuscitation shock (defined as need for continuous norepinephrine or epinephrine infusion to maintain mean arterial pressure above 60 mmHg, over 6 h after OHCA [28]), use of targeted temperature management, and use of veno-arterial ECMO for refractory cardiac arrest.

Additional data about organ donation were collected retrospectively by two independent blinded investigators. The kappa coefficients indicated excellent agreement between these investigators (0.87 to 1.00).

In France, potential donors with brain death are registered in the national information system (CRISTAL) run by the *Agence de la Biomedecine* [29]. Demographic, clinical, and laboratory data are collected prospectively by specialized organ-donation coordinators during the care of brain-dead patients. Authorization of organ donation must be obtained from the next of kin. In this project, CRISTAL database was used to retrieve long-term outcome of transplant and recipients. Graft survival was defined as a patient alive at 1-year follow-up, with a functional graft.

### Outcome measures

The primary outcome was organ donation after confirmed brain death. Secondary outcomes were eligibility for organ donation, reasons for absence of organ donation in eligible patients, number of retrieved and transplanted organs, and 1-year graft survival.

### Statistical analysis

Continuous variables were described as median [interquartile range] and qualitative variables as number (%). Linearity of quantitative variables was assessed using fractional polynomial regression. Non-linear continuous variables were dichotomized according to the median. Comparisons were done with Pearson Chi-square test or Fisher test, as appropriate, for categorical variables and Student’s* t* test or the Wilcoxon–Mann–Whitney test, as appropriate, for continuous variables.

Variables associated with organ donation with *P* values below 0.20 by univariate analysis were entered into a multivariate logistic regression model then selected by backward stepwise elimination. When a qualitative interaction between variables was found, stratification on appropriate variables was performed. Model discrimination was evaluated by computing the area under the receiver operating characteristic curve. Patients with missing data were excluded from the main analysis. A sensitivity analysis was performed using multiple imputation by chained equations: 20 datasets were created, with missing values replaced by imputed values. The results of the analyses of individual imputed datasets were combined according to Rubin rules. We also performed a sensitivity analysis including calendar year as a covariable to consider a potential temporal trend.

Graft survival was assessed by plotting Kaplan–Meier curves. Predictors of graft survival at 1 year were identified using Cox univariate analysis and the log-rank test.

All tests were two-sided, with *P* values below 0.05 being taken to indicate statistically significant differences. The analyses were run using STATA16.1 software (StataCorp, College Station, TX).

## Results

### Patients

**Fig. 1:**
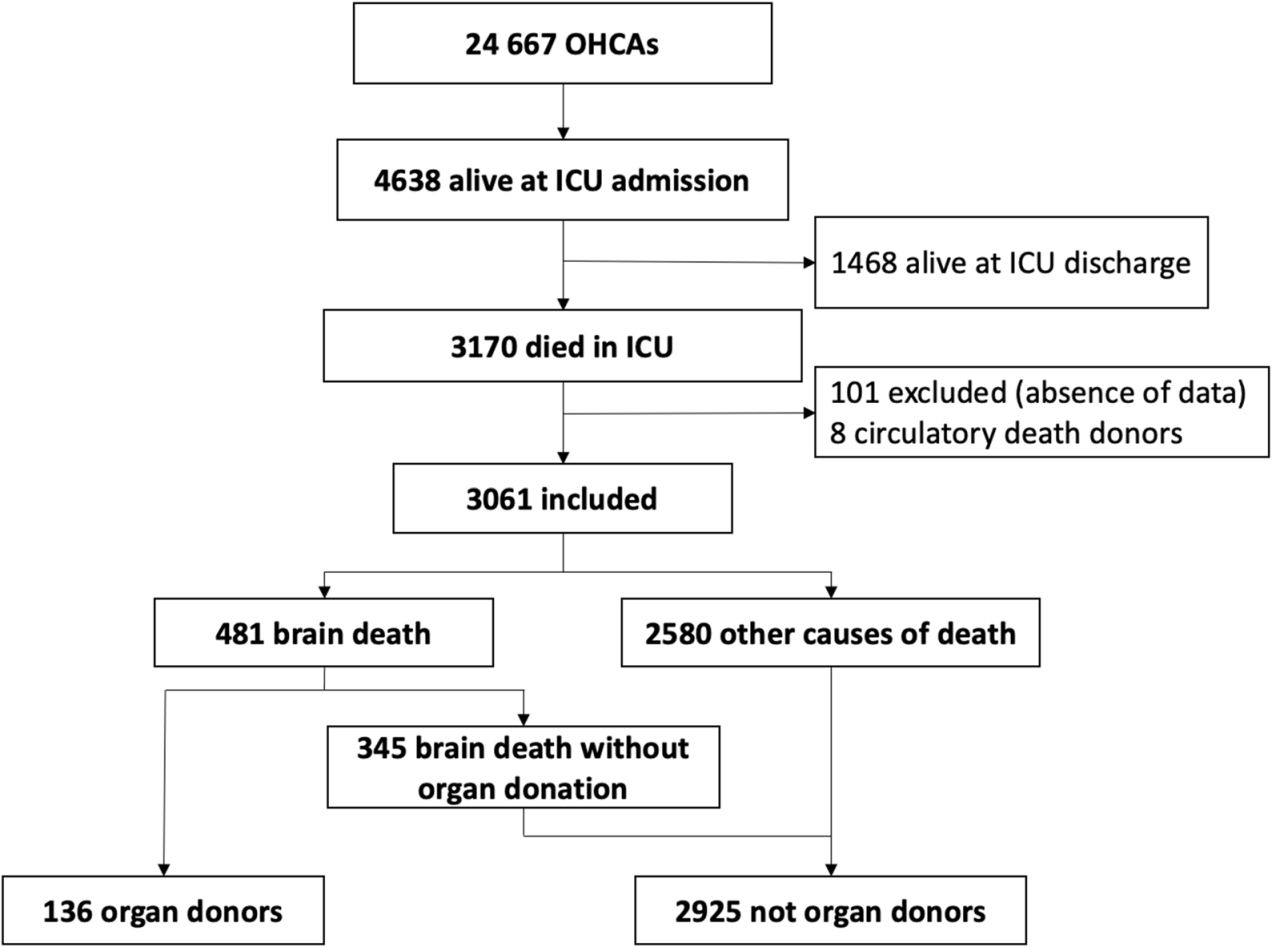
Patient flowchart. ICU: intensive care unit; OHCA: out-of-hospital cardiac arrest

Over the 7.5-year period, 4638 patients were admitted alive to ICUs after OHCA. Among them, 3170 died in the ICU and were screened for the study (Fig. 1), including 3061 who were enrolled. Of the enrolled patients, 481 (16%) experienced brain death, including 136 who donated organs. The organ donors represented 2.9% (95% confidence interval [95% CI], 2.4–3.4%) of patients admitted alive and 4.4% (95% CI 3.7–5.2%) of patients included in our study.

Table 1 reports the main patient characteristics. Organ donors were significantly younger (54 vs 64 years, *P* < 0.001) with more OHCA of neurological cause (39% vs 5%, *P* < 0.001) than patients without organ donation. Delays from OHCA to CPR and from CPR to ROSC did not significantly differ between groups.

**Table 1:** Utstein characteristics of patients with out-of-hospital cardiac arrest

	Organ donors (*n* = 136)	Not organ donors (*n* = 2925)	*P* value
Age, years, median [IQR]	54 [48–64]	64 [54–75]	< 0.0001
Males, n (%)	82 (60)	1966 (67)	0.09
Neurological cause of OHCA, n (%)	53 (39)	132 (5)	< 0.001
OHCA at home, n (%)	80 (59)	1933 (66)	0.08
Bystander-witnessed, n (%)	119 (87)	2549 (87)	0.92
Bystander CPR, n (%)	82 (69)	1775 (69)	0.91
Shockable rhythm, n (%)	38 (29)	1045 (38)	0.05
Epinephrine given, mg, median [IQR]	2 [1–4]	3 [1–5]	0.06
No-flow time, min, median [IQR]	5 [0–10]	5 [0–10]	0.64
Low-flow time, min, median [IQR]	27 [17–43]	26 [17–40]	0.86
ICU in transplant center, n (%)	111 (82)	2092 (71)	0.01
VA-ECMO for refractory OHCA, n (%)	22 (16)	372 (13)	0.24
Admission creatinine, µmol/L, median [IQR]	109 [88–125]	127 [99–165]	< 0.001
Admission pH, median [IQR]	7.18 [7.10–7.31]	7.16 [7.02–7.27]	< 0.001
Targeted temperature management, n (%)	61 (45)	1394 (48)	0.47
Post-resuscitation shock, n (%)	71 (61)	2041 (76)	< 0.001

CPR: cardiopulmonary resuscitation; OHCA: out-of-hospital cardiac arrest; IQR: interquartile range; VA-ECMO: veno-arterial extracorporeal membrane oxygenation

### Brain death and organ donation

**Fig. 2:**
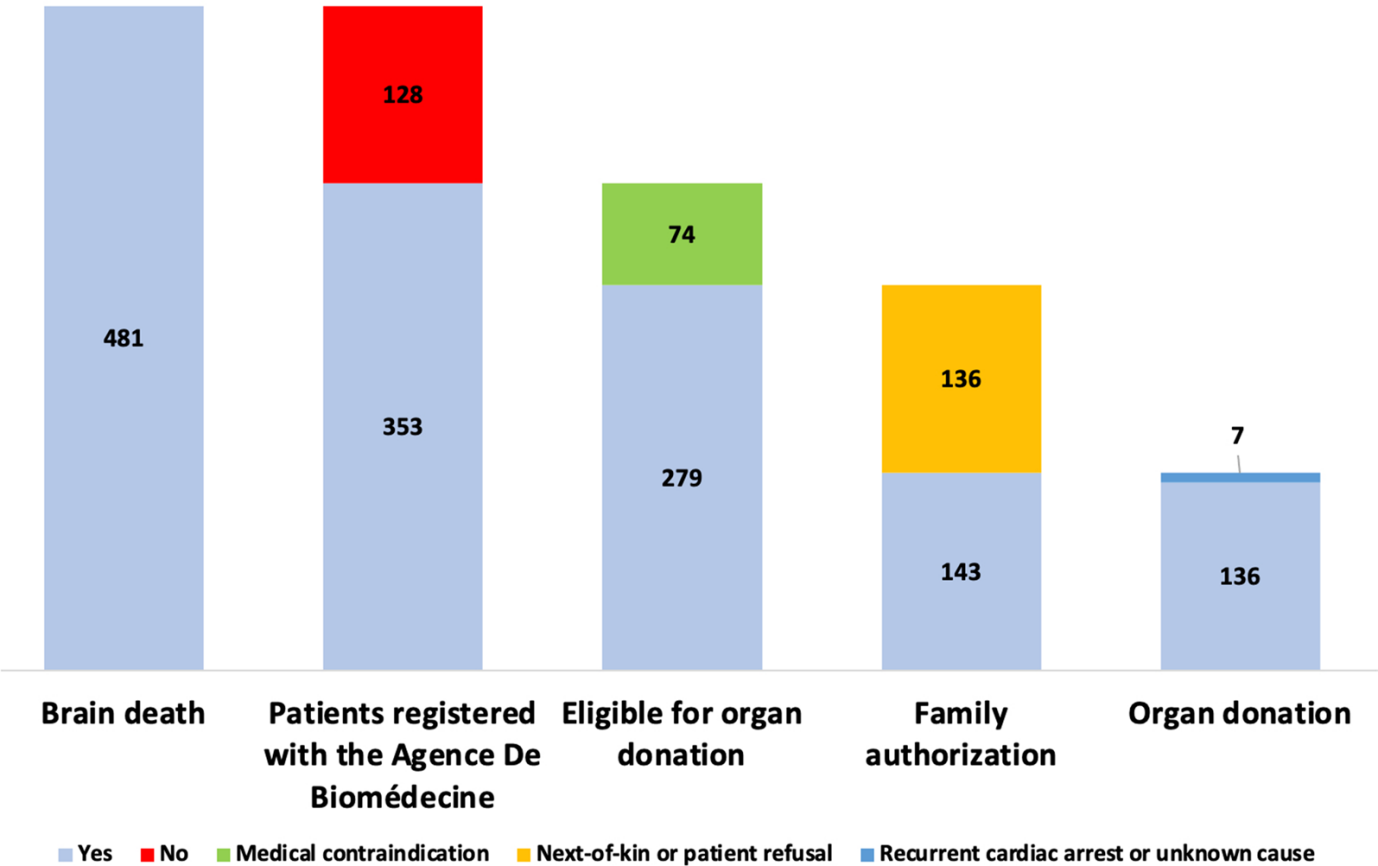
Process from brain death to organ donation

Of the 481 patients with brain death, 353 (73%) were reported to the *Agence de la Biomédecine*, including 279 deemed eligible for organ donation (Fig. 2). The main reasons for noneligibility were severe multiorgan failure (34%), known active malignancy (26%), and active infection (16%). Among the 279 eligible patients, 136 had opted out of organ donation or had refusal of organ donation by family members. Finally, 136 patients donated organs, i.e., 28% of patients with brain death. In all, 206 kidneys, 85 livers, and 24 hearts were transplanted. The median number of organs donated and transplanted per donor was 2.9 and 2.6, respectively.

### One-year graft survival

**Fig. 3:**
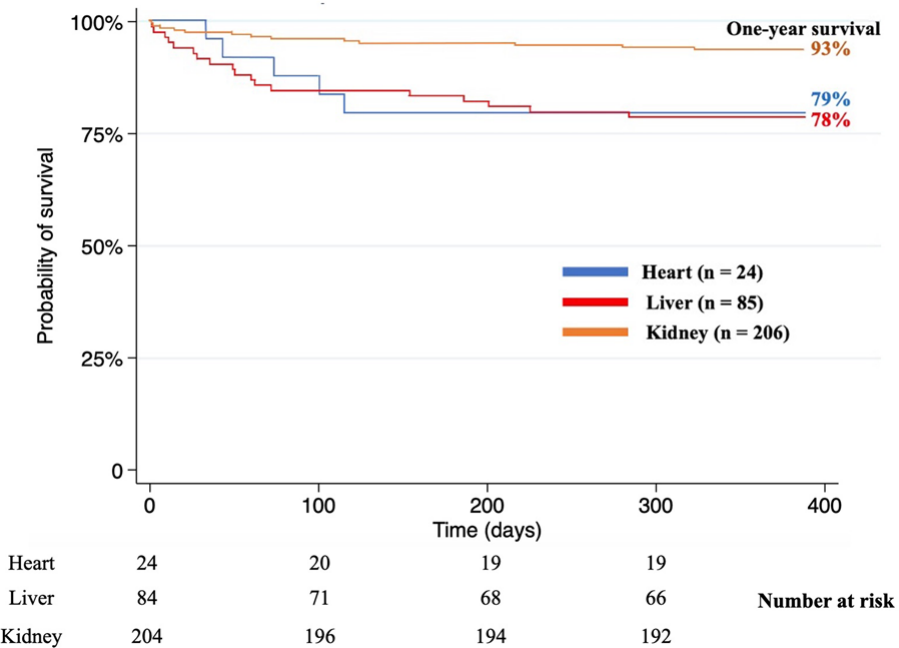
Graft survival from donors with brain death after out-of-hospital cardiac arrest

Figure 3 shows the 1-year outcomes: graft survival rates were 93% for kidneys, 79% for hearts, and 78% for livers.

Table 2 lists the Utstein characteristics of patients according to 1-year graft survival. Overall, no characteristic was associated with these outcomes.

**Table 2:** Univariate analysis of variables associated with 1-year graft survival

Variable	HR	95% CI	*P* value (log-rank test)
Donor age ≥ 54 years	0.99	[0.32 - 3.02]	0.98
Neurological cause of OHCA	1.11	[0.37–3.31]	0.85
Bystander-witnessed	0.75	[0.17–3.41]	0.72
Shockable rhythm	0.54	[0.07–4.16]	0.55
No-flow time ≥ 5 min	2.03	[0.53–7.86]	0.29
Low-flow time ≥ 27 min	0.97	[0.28–3.34]	0.96
≥ 3 mg epinephrine administered	0.41	[0.11–1.52]	0.17
Post-resuscitation shock	1.26	[0.38–4.19]	0.70

HR: hazard ratio; OHCA: out-of-hospital cardiac arrest

### Factors associated with organ donation

**Fig. 4:**
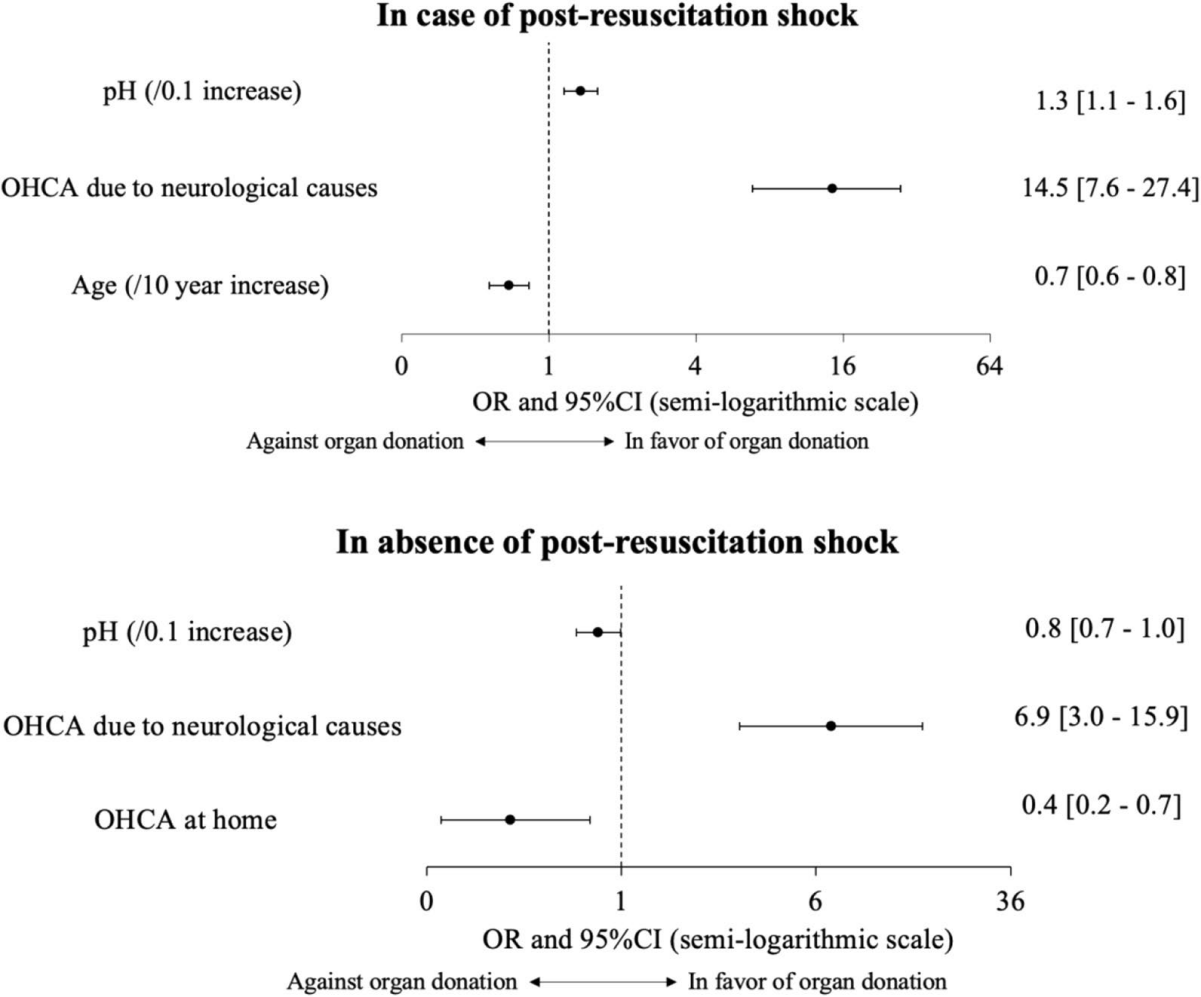
Significant independent predictors of organ donation in patients with and without post-resuscitation shock. Only patients without missing data were included; 95% CI: 95% confidence interval; OHCA: out-of-hospital cardiac arrest; OR: odds ratio

An interaction between post-resuscitation shock and pH was identified, and the main analysis was therefore stratified on post-resuscitation shock (Fig. 4). In patients with post-resuscitation shock, factors independently associated with organ donation were neurological cause of OHCA (odds ratio [OR], 14.5; 95% CI 7.6–27.4; *P* < 0.001) and higher pH (OR/0.1 increase, 1.3; 95% CI 1.1–1.6; *P* < 0.001), whereas older age was negatively associated (OR/10-year increase, 0.7; 95% CI 0.6–0.8; *P* < 0.001). In patients without post-resuscitation shock, neurological cause of OHCA was independently associated with organ donation (OR, 6.9; 95% CI 3.0–15.9; *P* < 0.001), whereas higher pH (OR, 0.8; 95% CI 0.7–1.0; *P* = 0.04) and OHCA at home (OR, 0.4; 0.2–0.7; *P* = 0.006) were negatively associated with organ donation. Pseudo-R2 of the model was 0.17.

The multiple-imputation sensitivity analysis produced similar results (Additional file 1: Table S1). Sensitivity analysis adjusted for calendar year did not reveal any temporal trend.

## Discussion

In this large population-based study over 7.5 years, 4.4% of patients who died in the ICU after OHCA became organ donors. Neurological cause of OHCA was independently associated with organ donation in patients with and without post-resuscitation shock. In patients with post-resuscitation shock, higher pH at ICU admission was also associated, whereas older age was negatively associated with donation. In the group without post-resuscitation shock, both higher pH and OHCA at home were negatively associated with donation. Importantly, 1-year graft survival was high and did not differ according to Utstein characteristics. Thus, patients with brain death after OHCA appear to be suitable candidates for organ donation and could be identified early during the ICU course.

A 2016 meta-analysis showed that 13% of patients who died after OHCA developed brain death and that, among these, 42% (6% of all deceased patients) became organ donors [6]. This result was consistent with previous studies [7–10, 15, 28, 30, 31]. In our study, the corresponding proportions were 16% and 28%. Overall, 4% of patients who died in ICU after OHCA donated organs. This lower proportion is consistent with our population-based study design, which contrasts with the transplant center-based recruitment used in most of the previous studies. Thus, we included all patients with OHCA in a well-defined geographic area.

Several factors were significantly and independently associated with organ donation. A neurological cause of OHCA was strongly associated with organ donation in patients with or without post-resuscitation shock. A previous study found that brain death developed in most patients with OHCA due to neurological causes [26]. In the group with post-resuscitation shock, higher pH was associated with organ donation, perhaps due to an association linking acidosis to multiorgan failure, a contraindication to organ donation [32]. In patients with shock whose pH is low, a higher pH is associated with organ donation, and might be desirable. By contrast, in patients without shock, whose pH is normal, a higher pH was not associated with organ donation. Older age was associated with absence of organ donation in this group, in keeping with earlier data [15, 16]. Younger patients are less likely to have comorbidities contraindicating organ donation [33]. In the group without post-resuscitation shock, higher pH and OHCA at home were associated with absence of organ donation. OHCA at home is less likely to be witnessed, resulting in a longer no-flow time, with greater organ damage.

In our population, a quarter of patients with brain death became organ donors. An evaluation of the reasons for not donating organs may indicate means of improving this disappointingly low proportion. A fourth of patients with brain death were not registered with the *Agence de Biomedecine*. Identifying the reasons of absence of registration could be a lever against organ shortage. About half the patients with brain death had either opted out of organ donation, in compliance with French law, or had relatives who were unwilling to accept that they become organ donors. Similar proportions have been reported in the US and UK. In Spain, which also has an opt-out system, only about 15% of relatives refuse organ donation [8, 15, 16, 34]. These reasons for lack of organ donation among brain deaths might explain partly the low value of pseudo-R2 of our model.

In our study, the medians per patient were 2.9 donated organs and 2.6 transplanted organs, in agreement with earlier data [8, 15]. One-year transplant survival rates were in line with data from non-OHCA donors: 79% vs. 77% (95% CI 76–78.1%) for hearts, 93% vs. 92% (95% CI 91.5–92%) for kidneys, and 78% vs 87.1% (95% CI 86.6–87.7%) for livers [35]. In previous studies of donors after cardiac arrest, long-term transplant outcomes were similar to those seen with other donor types [10–12]. These encouraging long-term outcomes underline the potential value of patients with OHCA as organ donors. Moreover, in our study, Utstein characteristics were not associated with 1-year graft survival. This finding suggests that organ donation should be considered for all patients with brain death after OHCA, regardless of cardiac arrest characteristics.

Our study has several strengths. We performed a multivariate analysis to identify independent predictors of organ donation, whereas several other studies used only univariate analysis [15]. Our population-based design with recruitment in a large geographic area where the management of OHCA is standardized provides greater representativity compared to single-center studies. We included only patients with OHCA, a specific form of cardiac arrest exhibiting marked differences from in-hospital cardiac arrest, although some studies pooled the two [16]. Finally, we assessed the 1-year outcomes of the transplanted organs. Nonetheless, we must acknowledge several limitations. Data were missing for some variables. However, the findings from the sensitivity analysis using multiple imputation were similar to those of the main analysis. We did not have a control group of non-OHCA donors for a comparison of 1-year transplant survival. The only information we had on the admitting hospitals was status as a transplant or non-transplant center, and we cannot rule out residual confounding related to other hospital characteristics. However, a recent study done in France in patients with OHCA found no difference in patient survival across three categories of admitting hospitals differentiated by cardiac arrest case load and catheterization laboratory availability [36]. Studies of associations linking hospital features to organ donation from patients with OHCA might produce useful information. Furthermore, inclusion of patients during a 7-year period could introduce a variability in the likelihood of referral for organ donation, due to practice changes. However, exploratory analysis regarding temporal trends did not report any association between calendar year and organ donation. Finally, some variables, such as socioeconomic data, were not available.

## Conclusions

In our study, 4% of patients who died in the ICU after OHCA donated organs after brain death. This low proportion invites greater attention to the possibility of organ donation by patients admitted to the ICU with ROSC after OHCA, notably those with neurological conditions, younger age, and less acidosis. The similar 1-year graft survival to those seen with non-OHCA donors further supports such attention. Outcomes did not differ across Utstein characteristics. Efforts are also needed to increase the proportion of relatives willing to accept organ donation by their loved one. Patients with OHCA constitute a valuable source of donated organs.

## Supplementary Information

**Additional file 1. **Paris Sudden Death Expertise Center Investigators list.

## Data Availability

The datasets generated and/or analyzed during the current study are not publicly available due to confidentiality, but are available from the corresponding author on reasonable request.

## Abbreviations

CI: Confidence interval
CPR: Cardiopulmonary resuscitation
ICU: Intensive care unit
OHCA: Out-of-hospital cardiac arrest
OR: Odds ratio
ROSC: Return of spontaneous circulation
SDEC: Sudden Death Expertise Center (Paris, France)
VA-ECMO: Veno-arterial extracorporeal membrane oxygenation
