# Supplementary material for: Organ donation after out-of-hospital cardiac arrest: a population-based study of data from the Paris Sudden Death Expertise Center
Source: Ann Intensive Care. 2022 Jun 6;12:48. doi: 10.1186/s13613-022-01023-7 (PMC9170852; doi:10.1186/s13613-022-01023-7)
Supplement: Supplementary file 1 — Additional file 1. Paris Sudden Death Expertise Center Investigators list. [file 13613_2022_1023_MOESM1_ESM.docx]

**Supplementary Material**

**Table S1:** Predictors of organ donation after stratification on post-resuscitation shock (missing data handled by multiple imputation)

| **Variable** | **OR** | **95%CI** | ***P* value** |
| --- | --- | --- | --- |
| **Group with post-resuscitation shock** | | | |
| Neurological cause of OHCA | 14.1 | [8.1–24.4] | <0.001 |
| Age (per 10-year increase) | 0.7 | [0.6–0.8] | <0.001 |
| pH (per 0.1 increase) | 1.4 | [1.2–1.6] | <0.001 |
| **Group without post-resuscitation shock** | | | |
| Neurological cause of OHCA | 6.9 | [3.5–13.3] | <0.001 |
| Age (per 10-year increase) | 0.7 | [0.6–0.9] | 0.002 |

Legend: OR, odds ratio; 95%CI, 95% confidence interval; OHCA, out-of-hospital cardiac arrest

**Missing data in each variable of interest**

|  | **Missing data, n(%)** |
| --- | --- |
| Age, years, median [IQR] | 0 (0) |
| Males, n (%) | 0 (0) |
| Neurological cause of OHCA, n (%) | 1 (<1) |
| OHCA at home, n (%) | 4 (<1) |
| Bystander-witnessed, n (%) | 2 (<1) |
| Shockable rhythm, n (%) | 170 (6) |
| Epinephrine given, mg, median [IQR] | 65 (2) |
| ICU in transplant center, n (%) | 0 (0) |
| VA-ECMO for refractory OHCA, n (%) | 0 (0) |
| Admission creatinine, µmol/L, median [IQR] | 683 (22) |
| Admission pH, median [IQR] | 560 (18) |
| Targeted temperature management, n (%) | 43 (1) |
| Post-resuscitation shock, n (%) | 241 (8) |

**List of Paris Sudden Death Expertise Center investigators**

**Adnet F**, AP-HP, Paris 13 University, Avicenne Hospital, Emergency Medical Services (SAMU) 93, Bobigny, France

**Agostinucci JM**, AP-HP, Paris 13 University, Avicenne Hospital, Emergency Medical Services (SAMU) 93, Bobigny, France

**Aissaoui-Balanant N**, AP-HP, Georges Pompidou European Hospital, Medical Intensive Care Unit, Paris, France

**Algalarrondo V,** AP-HP, Antoine Beclere University Hospital, Department of Cardiology, Clamart, France

**Alla F**, French National Health Insurance (CNAMTS), Paris, France

**Alonso C**, Clinique Ambroise Paré, Department of Cardiology, Neuilly, France

**Amara W**, Hospital Group of Montfermeil – Le Raincy, Department of Cardiology, Montfermeil, France

**Annane D**, AP-HP, INSERM U1173 (Laboratory of Inflammation and Infection), University of Versailles - Saint Quentin en Yvelines, Raymond Poincare Hospital, General Intensive Care Unit, Garches, France

**Antoine C**, Biomedecine agency, Saint-Denis-la-Plaine, France

**Aubry P**, AP-HP, Bichat Hospital, Department of Cardiology, Paris, France

**Azoulay E**, AP-HP, Paris Diderot Sorbonne University, Saint Louis Hospital, Medical Intensive Care Unit, Paris, France

**Beganton F**, INSERM U970 - PARCC, Paris, France

**Billon C**, AP-HP, Georges Pompidou European Hospital, Department of Genetic, Paris, France

**Bougouin W**, Private Hospital Jacques Cartier, Department of xxx, Massy, France

**Boutet J**, AP-HP, Raymond Poincare Hospital, Emergency Medical Services (SAMU) 92, Garches, France

**Bruel C**, Saint Joseph Hospital, Medical-Surgical Intensive Care Unit, Paris, France

**Bruneval P**, AP-HP, Georges Pompidou European Hospital, Pathology Department, Paris, France

**Cariou A**, AP-HP, Cochin Hospital, Medical Intensive Care Unit, Paris, France

**Carli P**, AP-HP, Paris Descartes University, Necker - Enfants Malades Hospital, Emergency Medical Services (SAMU) 75, Intensive Care Unit, Paris, France

**Casalino E**, AP-HP, Emergency Medical Services (SAMU) 92, Clichy, France

**Cerf C**, Foch Hospital, Intensive Care Unit, Suresnes, France

**Chaib A**, Andre Gregoire Hospital, Department of Cardiology, Montreuil, France

**Cholley B**, AP-HP, Georges Pompidou European Hospital, Department of Anaesthesia and Surgical Intensive Care Medicine, Paris, France

**Cohen Y**, AP-HP, Avicenne Hospital, Medical-Surgical Intensive Care Unit, Bobigny, France

**Combes A**, AP-HP, Pitié-Salpetriere Hospital, Cardiology Institute (ICAN), Intensive Care Unit, Paris, France

**Coulaud JM**, CHI Montfermeil, Intensive Care Unit, France

**Crahes M**, AP-HP, Georges Pompidou European Hospital, Pathology Department, Paris, France

**Da Silva D**, Delafontaine Hospital, Intensive Care Unit, Saint-Denis, France

**Das V**, Andre Gregoire Hospital, Medical-Surgical Intensive Care Unit, Montreuil, France

**Demoule A,** AP-HP, Pitie Salpetriere Hospital, Medical Intensive Care Unit and Respiratory Division, Paris, France

**Denjoy I,** AP-HP, Bichat Hospital, Department of Cardiology, Paris, France

**Deye N**, AP-HP, Lariboisiere Hospital, Intensive Care Unit, Paris, France

**Diehl JL**, AP-HP, Georges Pompidou European Hospital, Medical Intensive Care Unit, Paris, France

**Dinanian S**, AP-HP, Antoine Beclere Hospital, Department of Cardiology, Clamart, France

**Domanski L**, Brigade de Sapeurs Pompiers de Paris (BSPP), Paris, France

**Dreyfuss D**, AP-HP, Louis Mourier Hospital, Intensive Care Unit, Colombes, France

**Duboc D**, AP-HP, Cochin Hospital, Department of Cardiology, Paris, France

**Dubois-Rande JL**, AP-HP, Henri Mondor Hospital, Department of Cardiology, Creteil, France

**Dumas F**, AP-HP, Cochin Hospital, Department of Emergency, Paris, France

**Duranteau J**, AP-HP, Bicetre Hospital, Department of Anaesthesia and Intensive Care Medicine, Le Kremlin-Bicetre, France

**Empana JP**, INSERM U970 - PARCC, Paris, France

**Extramiana F**, AP-HP, Bichat Hospital, Department of Cardiology, Paris, France

**Fagon JY**, AP-HP, Georges Pompidou European Hospital, Department of xxx, Paris, France

**Fartoukh M**, AP-HP, Sorbonne University (Paris 6), Tenon Hospital, Intensive Care Unit, Paris, France

**Fieux F**, CHI Montfermeil, Intensive Care Unit, France

**Gabbas M**, French National Health Insurance (CNAMTS), Paris, France

**Gandjbakhch E**, AP-HP, Pitie-Salpetriere Hospital, Cardiology Institute (ICAN), Department of Cardiology, Paris, France

**Geri G,** AP-HP, Ambroise Pare Hospital, Intensive Care Unit, Boulogne-Billancourt, France

**Guidet B**, AP-HP, Sorbonne University (Pierre et Marie Curie University – Paris 6), Saint Antoine Hospital, Intensive Care Unit, Paris, France

**Halimi F**, Private Hospital of Parly 2, Department of Cardiology, Le Chesnay, France

**Henry P**, AP-HP, Sorbonne Paris Cite University (Paris Diderot University), Lariboisiere Hospital, Department of Cardiology, Paris, France

**Hidden Lucet F**, AP-HP, Pitie-Salpetriere Hospital, Cardiology Institute (ICAN), Department of Cardiology, Paris, France

**Jabre P**, INSERM U970 – PARCC, Emergency Medical Services (SAMU) 75, Paris, France

**Joseph L**, Bicetre Hospital, Transplant Coordination, Le Kremlin-Bicetre, France

**Jost D**, Brigade de Sapeurs Pompiers de Paris (BSPP), Paris, France

**Jouven X**, AP-HP, Georges Pompidou European Hospital, Department of Cardiology, Paris, France

**Karam N**, AP-HP, Georges Pompidou European Hospital, Department of Cardiology, Paris, France

**Kassim H**, INSERM U970 - PARCC, Paris, France

**Lacotte J**, Private Hospital Jacques Cartier, Department of Cardiology, Massy, France

**Lahlou-Laforet K**, AP-HP, Georges Pompidou European Hospital, Department of Psychiatry, Paris, France

**Lamhaut L**, AP-HP, Paris Descartes University, Necker - Enfants Malades Hospital, Emergency Medical Services (SAMU) 75, Intensive Care Unit, Paris, France

**Lanceleur A**, Foch Hospital, Intensive Care Unit, Suresnes, France

**Langeron O**, AP-HP, Pitie-Salpetriere Hospital, Department of Anaesthesia and Surgical Intensive Care Medicine, Paris, France

**Lavergne T**, AP-HP, Georges Pompidou European Hospital, Department of Cardiology, Paris, France

**Lecarpentier E**, AP-HP, Henri Mondor Hospital, Emergency Medical Services (SAMU) 94, Creteil, France

**Leenhardt A**, AP-HP, Bichat Hospital, Department of Cardiology, Paris, France

**Lellouche N**, AP-HP, Henri Mondor Hospital, Department of Cardiology, Creteil, France

**Lemiale V**, AP-HP, Paris Diderot University, Saint Louis Hospital, Medical Intensive Care Unit, Paris, France

**Lemoine F**, Brigade de Sapeurs Pompiers de Paris (BSPP), Paris, France

**Linval F**, AP-HP, Paris 13 University, Avicenne Hospital, Emergency Medical Services (SAMU) 93, Bobigny, France

**Loeb T**, AP-HP, Raymond Poincare Hospital, Emergency Medical Services (SAMU) 92, Garches, France

**Ludes B**, Institute of Legal Medicine, Paris Descartes University, Paris, France

**Luyt CE**, AP-HP, Pitie-Salpetriere Hospital, Cardiology Institute (ICAN), Intensive Care Unit, Paris, France

**Maltret A**, AP-HP, Necker - Enfants Malades Hospital, Department of Pediatric Cardiology, Paris, France

**Mansencal N**, Ambroise Pare Hospital, Department of Cardiology, Boulogne-Billancourt, France

**Mansouri N**, AP-HP, Henri Mondor Hospital, Emergency Medical Services (SAMU) 94, Creteil, France

**Marijon E**, AP-HP, Georges Pompidou European Hospital, Department of Cardiology, Paris, France

**Marty J,** AP-HP, Henri Mondor Hospital, Emergency Medical Services (SAMU) 94, Creteil, France

**Maury E**, AP-HP, Sorbonne University (Pierre et Marie Curie University – Paris 6), Saint Antoine Hospital, Intensive Care Unit, Paris, France

**Maxime V**, AP-HP, Raymond Poincare Hospital, General Intensive Care Unit, Garches, France

**Megarbane B**, AP-HP, Lariboisiere Hospital, Intensive Care Unit, Paris, France

**Mekontso-Dessap A**, AP-HP, DHU A-TVB, CARMAS Research Group, Henri Mondor Hospital, Intensive Care Unit, Creteil, France

**Mentec H**, Victor Dupouy Hospital, Intensive Care Unit, Argenteuil, France

**Mira JP**, AP-HP, Cochin Hospital, Medical Intensive Care Unit, Paris, France

**Monnet X**, AP-HP, Bicetre Hospital, Medical Intensive Care Unit, Le Kremlin-Bicetre, France

**Narayanan K**, INSERM U970 - PARCC, Paris, France

**Ngoyi N**, AP-HP, Paris 7 University, Beaujon Hospital, Emergency Medical Services (SAMU) 92, Clichy, France

**Perier MC**, INSERM U970 - PARCC, Paris, France

**Piot O**, Centre Cardiologique du Nord, Department of Cardiology, Saint-Denis, France

**Pirracchio R**, AP-HP, Georges Pompidou European Hospital, Intensive Care Unit, Paris, France

**Plaisance P,** Emergency Medical Services (SAMU) 75, Lariboisière SMUR, Paris, France

**Plaud B**, AP-HP, Saint Louis Hospital, Department of Anaesthesia and Surgical Intensive Care Medicine, Paris, France

**Plu I**, AP-HP, Pitie-Salpetriere Hospital, Neuropathology Escourolle, Paris, France

**Raphalen JH,** AP-HP, Necker - Enfants Malades Hospital, Intensive Care Unit, Paris, France

**Raux M,** AP-HP, Pitié Salpétrière Hospital, Department of Anesthesiology and Critical Care, Paris, France

**Revaux F**, AP-HP, Henri Mondor Hospital, Emergency Medical Services (SAMU) 94, Creteil, France

**Ricard JD**, AP-HP, Louis Mourier Hospital, Intensive Care Unit, Colombes, France

**Richard C**, AP-HP, Bicetre Hospital, Medical Intensive Care Unit, Le Kremlin-Bicetre, France

**Riou B**, AP-HP, Pitie-Salpetriere Hospital, Sorbonne University (Pierre et Marie Curie University – Paris 6), Department of Emergency Medicine and Surgery, Paris, France

**Roussin F**, AP-HP, Saint Louis Hospital, Department of Anaesthesia and Surgical Intensive Care Medicine, Paris, France

**Santoli F**, Robert Ballanger Hospital, Intensive Care Unit, Aulnay-sous-Bois, France

**Schortgen F**, Centre Hospitalier Intercommunal Créteil, Intensive Care Unit, Creteil, France

**Sharifzadehgan A**, AP-HP, Georges Pompidou European Hospital, Department of Cardiology, Paris, France

**Sharshar T**, AP-HP, Sainte Anne Hospital, Intensive Care Unit, Paris, France

**Sideris G**, AP-HP, Sorbonne Paris Cite University (Paris Diderot University), Lariboisiere Hospital, Department of Cardiology, Paris, France

**Similowski T,** AP-HP, Pitie-Salpetriere Hospital, Department of xxx, Paris, France

**Spaulding C**, AP-HP, Georges Pompidou European Hospital, Department of Cardiology, Paris, France

**Teboul JL**, AP-HP, Bicetre Hospital, Medical Intensive Care Unit, Le Kremlin-Bicetre, France

**Timsit JF**, AP-HP, Bichat Hospital, Medical Intensive Care Unit, Paris, France

**Tourtier JP**, Brigade de Sapeurs Pompiers de Paris (BSPP), Paris, France

**Tuppin P**, French National Health Insurance (CNAMTS), Paris, France

**Ursat C,** AP-HP, Raymond Poincare Hospital, Emergency Medical Services (SAMU) 92, Garches, France

**Varenne O**, AP-HP, Cochin Hospital, Department of Cardiology, Paris, France

**Vieillard-Baron A**, AP-HP, Ambroise Pare Hospital, Intensive Care Unit, Boulogne-Billancourt, France

**Voicu S**, AP-HP, Lariboisiere Hospital, Intensive Care Unit, Paris, France

**Wahbi K**, AP-HP, Cochin Hospital, Department of Cardiology, Paris, France

**Waldmann V**, AP-HP, Georges Pompidou European Hospital, Department of Cardiology, Paris, France
